# Cumulative sum learning curve analysis of tubularized incised plate repair for hypospadias: a study of a single surgeon with a single surgical procedure

**DOI:** 10.3389/fped.2024.1375345

**Published:** 2024-04-11

**Authors:** Jiaqiang Li, Jiaqian Zhang, Hongwang Diao, Zhuoyuan He, Shoulin Li, Jianchun Yin

**Affiliations:** Department of Pediatric Urology, Shenzhen Children's Hospital, Shenzhen, Guangdong, China

**Keywords:** hypospadias, tubularized incised plate, clinical competence, proficiency, cumulative sum analysis of learning curve

## Abstract

**Purpose:**

To ascertain the quantity of instances by which a single surgeon achieves competency and proficiency in using tubularized incised plate (TIP) technique for the repair of distal and mid-shaft hypospadias using the cumulative sum (CUSUM) analysis.

**Methods:**

We retrospectively evaluated patients with distal and mid-shaft hypospadias who were treated by a single surgeon between 2015 and 2021, using a single primary TIP technique with a de-epithelialized Byars flap. Data including type of hypospadias, age at surgery, curvature, operation time (OT), length of the reconstructed urethra, and postoperative outcomes were collected and assessed. CUSUM was used to assess the trends in OT and complication rate (CR) in order to generate the learning curve. The evolution of OT and CR can be divided into three phases: learning, competence, and proficiency.

**Results:**

CUSUM identified three phases in the learning curves of all TIP repairs. The median OT decreased from 135 min [interquartile range (IQR) = 125–155] to 92 min (IQR = 80–100) (*P *< 0.001), CR decreased from 28 (28%) to 8 (5.3%) (*P *< 0.001), and reoperations decreased from 15 (15.2%) to 4 (2.6%) (*P *< 0.001). According to the CUSUM learning curve, technical competency plateaued after the 99th case, and both OT and CR entered a significantly declining proficiency phase after the 231st case. Further, when the neourethral length exceeded the total average, total complications, urethrocutaneous fistula, and reoperations increased (*P *= 0.013, *P *= 0.006, and *P *= 0.028, respectively).

**Conclusions:**

Our study suggests that surgeons performing TIP repair may reach technical competency and achieve proficiency after operating on 99,231 cases, respectively. Moreover, the longer the neourethral length, the higher is the CR.

## Introduction

Hypospadias is a common congenital anomaly of the penis with an incidence of 5–50/10,000 births (1). Tubularized incised plate (TIP) urethroplasty was introduced by Snodgrass et al. in 1994 and is increasingly used to repair hypospadias with mild penile ventral curvatures (2, 3).

Originally designed for monitoring performance and quality in industrial settings, cumulative sum analysis (CUSUM) technique has become a popular technique for analyzing surgical learning curves in the medical field (4, 5). This method can describe and evaluate the entire process of a doctor's competency in terms of proficiency in surgical techniques.

In this study, a series of distal and mid-shaft TIP urethroplasties performed by a single surgeon in a single center were reviewed. By utilizing CUSUM, our objective was to establish a learning curve and delineate the requisite number of cases for attaining competency and proficiency in the repair of hypospadias using primary TIP urethroplasty with a de-epithelialized Byars. Accordingly, the surgeon's experience appears to be an important factor in the complications and outcomes of surgery, especially hypospadias surgery, which requires delicate manipulation. Therefore, we hypothesized that in early clinical practice, there will be a long operative time (OT) and a higher complication rate (CR), which will gradually decrease with an increase in the number of operations.

## Material and methods

The need of informed consent was approved by the Ethics Committee of Shenzhen Children's Hospital (2021033). This retrospective study included 381 patients who underwent primary hypospadias repair performed by a single surgeon (J.Y.) in the first 7 years of practice (January 2015 to December 2021). Further, the only inclusion criterion was patients underwent a primary TIP urethroplasty with a de-epithelialized Byars flap. Accordingly, patients with either forms of hypospadias, distal or mid-shaft were included in this study. Data pertaining to age at surgery, type of hypospadias, curvature, OT, length of the reconstructed urethra, and postoperative outcomes were collected and analyzed. All cases included were followed up before being enrolled in our study. The follow-up included several clinical visits by the surgeon (J.Y.) for at least 13 months after surgery. Surgical complications and the need for revision surgery were assessed during the follow-up period.

OT and CR served as the main evaluation indicators for the surgical outcomes and learning curve. In this regard, OT was defined as the time from the initiation of the skin incision to the completion of the procedure. Accordingly, the CR was calculated by dividing the number of complications by the overall case count. Urethral strictures, urethrocutaneous fistulas, glandular dehiscence, and unfavorable cosmesis were among the surgical complications taken into account.

In order to generate a plot depicting the learning curve, the previously described method of CUSUM analysis was performed (6, 7). In this regard, the application equation model of CUSUM-OT was as follows: The difference between the OT of the first patient and the mean OT of all patients was used to establish the CUSUM-OT for the initial patient. The previous patient's CUSUM-OT plus the cumulative discrepancy between the subsequent patient's OT and mean OT to determine the CUSUM-OT for the subsequent patient. By employing this approach, the cumulative sum for each patient was repeatedly extrapolated until the CUSUM-OT value of the last patient reached zero (6).

The learning curve based on surgical complications was determined using CUSUM-CR. In this regard, four parameters need to be set before assessing the learning curve. Accordingly, α (type I error rates) and β (type II error rates) were predetermined as 0.05 and 0.2, respectively. The acceptable outcome rate was established as p0 = 0.08, which stems from previous literature (8). Further, the unacceptable outcome rate was set at p1 = 0.5, based on a previous report, and reaching 50% was considered unacceptable (9). Further, a graph was configured combining the plots of CUSUM-CR and CUSUM-OT to evaluate the learning curve as previously described (10, 11). The learning curve was divided into three phases (10). In this regard, phase 1 was determined as the beginning of the first case with the peaks of CUSUM-CR plot and the CUSUM-OT plot. Phase 2 was defined as the period starting from the peak cases and extending until the conclusion of the plateau period. Phase 3 encompassed the cases occurring after the plateau until the end of the evaluation period. Descriptive statistical methods of frequencies and percentages were used to summarize the categorical data extracted from clinical variables. The median and interquartile range (IQR) were used to summarize the nonparametric continuous variables. To further evaluate the differences between case characteristics and outcomes between the three phases, we analyzed the categorical data using the Chi-square test and the continuous data using the Kruskal-Wallis *H*-test. Accordingly, *p*-values were generated by bivariate analysis. For small sample events, Fisher's exact test was used for statistical analysis. Additionally, the Bonferroni method was used to adjust *p*-values when conducting multiple comparisons. Statistical analysis was performed using the SPSS software (version 19.0; SPSS, Chicago, IL, USA). All methods were performed in accordance with the relevant guidelines and regulations. Statistical significance was set at *P* < 0.05.

### Surgical technique

The steps of the standard procedure were performed as we previously described (12). The only difference from the Snodgrass W.T. (3) is the modified method of barrier layer. Briefly, the prepuce was vertically incised into two halves and brought around the side of the penile ventral shaft. The end of one side of flap was de-epithelialized to harvest a barrier layer.

## Results

More than 2,500 hypospadias repair were performed at our institution during the 7-year study period. Accordingly, a single surgeon (JCY) operated on 381 of these patients using a primary TIP urethroplasty with a de-epithelialized Byars flap.

At the time of the initial surgery, the median age of the overall patient cohort was 43.3 months (IQR = 20.5–52.8). The median follow-up duration for all patients was 21.9 months and the minimum follow-up time was 13 months. In our cohort, 236 (61.9%) patients were presented with distal and 145 (38.1%) patients with midshaft hypospadias. Accordingly, 148 (38.9%) cases required penile curvature correction.

The median OT was 107.5 min (IQR = 93.2–122.4). The *P* value for the difference between the neourethral lengths of the three phases was >0.05. Of the 49 patients with complications, 21 (42.9%) had urethrocutaneous fistulas, 18 (36.7%) had unfavorable cosmesis, six (12.2%) had glanular dehiscence, and four (8.2%) had urethral strictures. Accordingly, 27 patients underwent reoperations, including all cases with urethrocutaneous fistulas and urethral stricture, and some cases who had glanular dehiscence (Table 1).

**Table 1:** Characteristics and outcome of the hypospadias patients.

Variables	Phase 1, *N* = 99	Phase 2, *N* = 131	Phase 3, *N* = 151	*P-*value	Phase 1 vs. Phase 2	Phase 2 vs. Phase 3	Phase 1 vs. Phase 3
Age (months)	49.1 (25.1–76.2)	46.7 (25.6–64.1)	36.8 (13.8–48.4)	**0** **.** **008**	0.52	**0** **.** **021**	**0** **.** **003**
Hypospadias subcategories				0.07	0.75	0.07	**0** **.** **045**
Distal	67 (67.7%)	86 (65.6%)	83 (55%)				
Mid-shaft	32 (32.3%)	45 (34.4%)	68 (45%)				
Curvature	46 (46.5%)	44 (33.6%)	58 (38.4%)	0.14	**0** **.** **048**	0.4	0.21
Operative time (min)	135 (125–155)	104 (90–115)	92 (80–100)	**<0** **.** **001**	**<0** **.** **001**	**<0** **.** **001**	**<0** **.** **001**
Neourethral length (mm)	13.0 (10.2–15.7)	13.3 (10.5–16.1)	13.9 (11.1–18.4)	0.4	0.68	0.32	0.23
Complications	28 (28%)	16 (12.2%)	8 (5.3%)	**<0** **.** **001**	**0** **.** **002**	**0** **.** **037**	**<0** **.** **001**
Urethrocutaneous fistula	10 (10.1%)	7 (5.3%)	4 (2.6%)	**0** **.** **041**	0.17	0.24	**0** **.** **012**
Glanular dehiscence	3 (3%)	2 (1.5%)	1 (0.7%)	0.34	0.44	0.48	0.14
Urethral stricture	3 (3%)	1 (0.7%)	0 (0%)	0.06	0.19	0.28	**0** **.** **031**
Unfavorable cosmesis	9 (9.1%)	6 (4.6%)	3 (2%)	**0** **.** **035**	0.17	0.22	**0** **.** **01**
Redo operations	15 (15.2%)	8 (6.1%)	4 (2.6%)	**<0** **.** **001**	**0** **.** **024**	0.15	**<0** **.** **001**

Statistically significant *p*-values are in bold.

The median neourethral length was 13.5 mm (IQR = 11.2–16.4). Accordingly, patients were divided into two groups: those with neourethral length <13.5 mm and those with neourethral length ≥13.5 (Table 2). The *P*-value for cosmesis outcome was >0.05. There was a significant difference between the groups considering complications (*P* = 0.013), urethrocutaneous fistula (*P* = 0.006), and reoperations (*P* = 0.028).

**Table 2:** Correlation between neourethral length and outcome of the hypospadias patients.

Variables	Neourethral length		*P-*value
	<13.5 mm	>13.5 mm	
Complication			**0.013**
Yes	20	29	
No	198	134	
Urethrocutaneous fistula		** **	**0** **.** **006**
Fistula	6	15	** **
No fistula	212	148	** **
Cosmesis			0.073
Unfavorable	11	7	** **
Favorable	207	156	** **
Redo operations			**0** **.** **028**
Yes	10	17	** **
No	208	146	** **

Statistically significant *p*-values are in bold.

**Figure 1:**
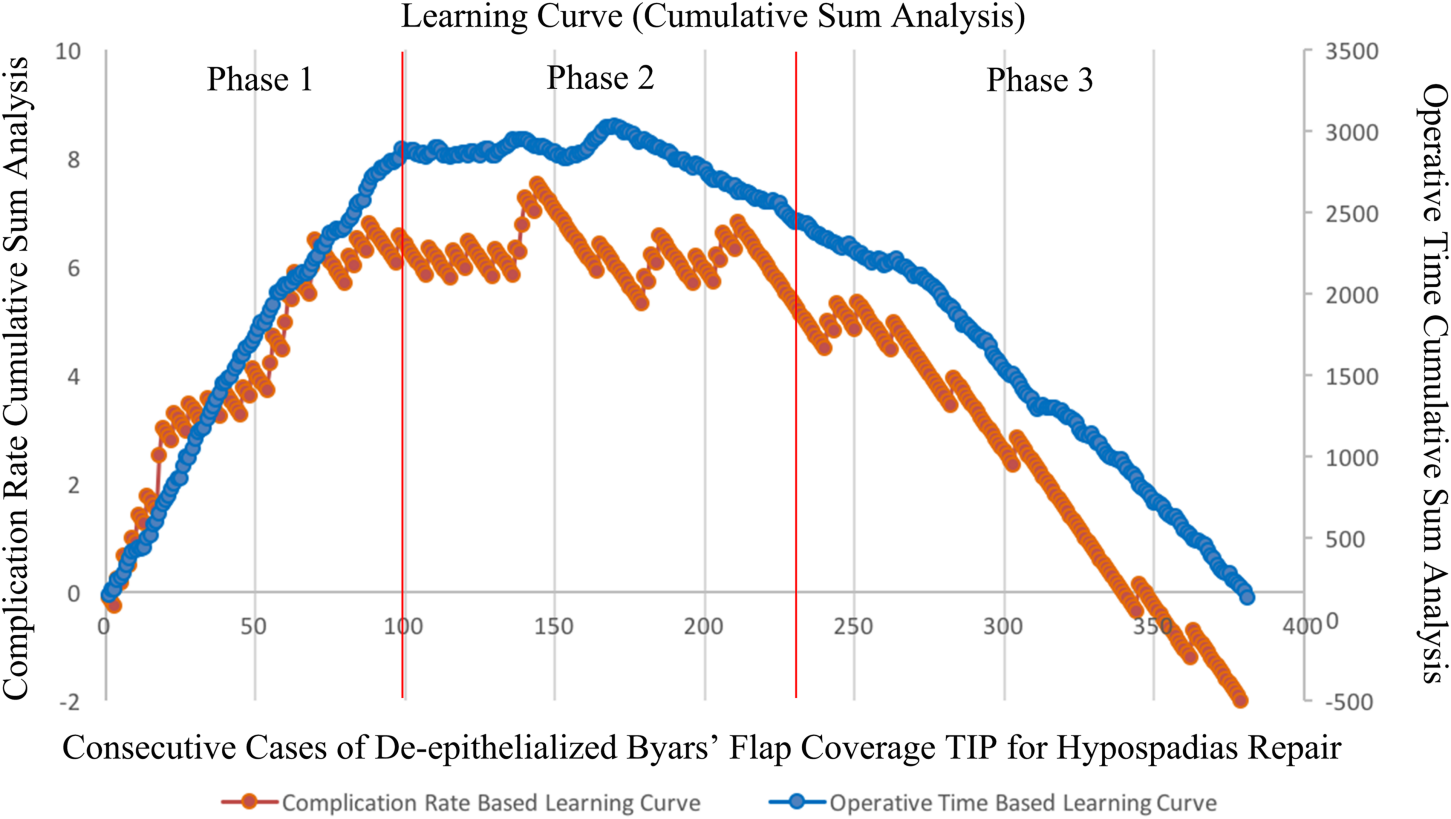
Learning curve of CUSUM plot for OT and CR.

Phase 1 was set from the start of the practice to the 99th case, Phase 2 was set from the 99th case to the 231st case, and Phase 3 was set after the 231th case (Figure 1). The *P*-value for median OT was <0.001 between the three phases (135 min [IQR = 125–155]; 104 min [IQR = 90–115]; 92 min [IQR = 80–100], respectively). Further, the CR was significantly different between the three phases (*P* < 0.001, total; phase 1 vs. phase 2, *P* = 0.002; phase 2 vs. phase 3, *P* = 0.037; phase 1 vs. phase 3, *P* < 0.001, respectively). The peaks for OT and CR were at the 167th and the 144th cases, respectively.

## Discussion

TIP is one of the most frequently used procedures for distal hypospadias repair, which its use is becoming more frequent also for mid-shaft and proximal cases (13, 14). Various surgical modifications have been applied to repair hypospadias (15, 16). De-epithelialized preputial skin flap coverage technique is an easy and valuable modified method for hypospadias repair (15, 16).

This study employed the CUSUM technique to analyze the experiential learning curve of a sole surgeon in repairing hypospadias using this modified surgical procedure, focusing on experiential analysis. The learning curve was divided into three phases. Accordingly, in the initial learning phase, not only there was a longer OT, but also a higher CR. After the competence phase, the proficiency phases of both the OT and CR decreased exponentially. As the surgeon's experience increased, both OT and CR notably declined, and the decrease in CR was mainly attributed to fewer fistulas and better cosmesis.

Recently, a single-surgeon series of CUSUM learning curves by Zu'bi et al. showed that the competence phase commenced after surpassing the 127th case, while the proficiency phase of both OT and CR was significantly decreased after the 234th case (10). In comparison, we performed the TIP repair for hypospadias with a modified overlay. Our study showed that the learning curve reached a competence phase after operating on 99 patients, and after a competence phase of 131 patients, the proficiency phase reached by the patient number 231. Both the competence and proficiency phases of our series were achieved earlier, but the competence phase was longer. Moreover, Zu'bi et al. reported a higher severity of hypospadias and younger patients being repaired using the TIP technique during the proficiency phase, resulting in a decreased incidence of complications. Our results also showed fewer complications in younger patients underwent repair using our modified surgical procedure. Further, Zu'bi et al. indicated that they used two types of coverage techniques (dartos layer and spongioplasty) to cover the urethra, which was notably increased in the proficiency phase, which may have influenced the statistical results to some extent. Our series used the single coverage technique for hypospadias repair, which reduces the bias.

Horowitz and Salzhauer (17) reported earlier learning curves for 231 single-stage hypospadias operations performed by one pediatric urologist (M.H.) over a 5 years period in practice. Further, in their study fistula rates were used as the only objective outcome measure for CR, and there was a significant decrease in fistula rates between the first and second two-years. The fistula rates showed a significant decline in each year of observation from 23% to 6%. The fistula rates in our series also showed a gradual decline from the learning phase (10.1%) to the proficiency phase (2.6%).

Numerous studies have confirmed the beneficial impact of surgeon experience on the success rate of hypospadias repair using the TIP technique (10, 18, 19). Rompre et al. (19) reported 303 cases who underwent primary TIP performed by a single surgeon. The learning curve stabilized after 50–70 cases and subsequently continued to decline further in a predictable negative exponential curve. Parikh et al. (18) retrospectively reviewed 184 patients who underwent TIP by a single fellowship-trained surgeon using CUSUM methodology. Their CR remained within the acceptable range until approximately 150 operations were performed, before the CR fell below the lower limit. They demonstrated that the fistula rate in distal hypospadias repairs fell below the acceptable rate after the initial 110 operations. All these studies supported this statement thar as the surgeon's experience increases, the complication rate decreases. Our series showed that even after the initial learning phase in 99 cases, the surgical outcomes improved constantly with time and experience.

Bush et al.'s (8) paper on distal hypospadias repair after pediatric urology fellowship training compared junior surgeons with their mentors to evaluate outcomes. The claim that juniors achieve similar outcomes as mentors can be attributed to several factors. One key factor could be the structured and intensive nature of pediatric urology fellowship training. Junior surgeons undergo focused training with a curriculum designed to enhance their skills and knowledge specific to hypospadias repair. This targeted training equips them with the necessary proficiency to achieve comparable outcomes to their experienced mentors. Moreover, the study may highlight the impact of dedicated supervision on surgical outcomes. Junior surgeons likely receive close mentorship and guidance from experienced practitioners during training and surgeries. This tighter supervision ensures that junior surgeons adhere to best practices, minimize errors, and effectively manage potential complications, thus contributing to comparable outcomes between juniors and mentors. Structured training plays a critical role in this context by providing a framework for skill development and progression. Structured training programs offer numerous benefits, including standardized skill acquisition, continuous learning opportunities, and a supportive environment for skill development. By following established protocols and guidelines, registrars can build confidence, enhance their surgical proficiency, and ultimately contribute to improved patient outcomes.

Although surgeon experience is an important factor in the success of hypospadias surgery, there are still many other factors that influence clinical outcomes, such as meatal location, glans size, and presence of curvature. The initial meatal position, which is the neourethral length that needs to be reconstructed, is the major factor influencing TIP outcomes. Rompre et al. (18) reported that the CR of non-distal hypospadias was higher than that of distal hypospadias. Kim et al. (20) and Lucas et al. (21) reported similar results. Previous studies have mainly explored the relationship between the types of hypospadias and complications, and few studies have reported a correlation between the length of specific defective urethra and complications. Our series results showed that the rates of total complications, urethrocutaneous fistula, and reoperations were lower in the group with neourethral length less than 13.5 mm, compared to that of the other group.

Urethrocutaneous fistula is the most frequent complication of hypospadias, occurring in 13% of patients according to a meta-analysis (22). Further, another large meta-analysis including 49 studies (4,675 patients) reported that only 5.7% of fistulas were observed after primary distal hypospadias repair (23). In our study, although the incidence of fistula was high in the learning phase (10.1%), it reached 2.6% in the proficiency phase, with an overall fistula rate of 5.5% (21/381). Accordingly, our fistula rate is similar to the result of the meta-analysis reported by Pfistermuller et al. (23); however, 38% (145/381) of our cases were mid-shaft hypospadias.

The influence of surgeon experience on the OT of TIP has been reported in many studies, which reported that OT continues to decrease as the surgeon's experience increases (10, 24). We demonstrated that the reduction in OT did not lead to an increase in CR but continued to decrease.

This study has several limitations. First, the retrospective design was conducted at a single center, which might result in a study bias. Second, there must be different follow-up periods for patients who underwent surgery in different phases. Additionally, some patients had only one follow-up visit at 13 months, which might have resulted in missing some complications. Third, our learning curve was formed by a single surgeon, and the others may have different learning patterns. However, we believe that this study has implications for surgeons who use this surgical technique, making it easier for them to quickly learn whether they are proficient in the procedure.

## Conclusion

TIP repair is a surgical technique requires attention to details and fine techniques. A certain number of cases is required to achieve technical competence and optimal outcomes. Early surgery is prone to complications, which will undermine the confidence of doctors to a certain extent. This study suggests that a surgeon may reach technical competency by the 99th case and achieve proficiency by the 231st case. Nevertheless, this retrospective study solely involving one surgeon highlights the necessity for prospective studies employing CUSUM analysis with multiple pediatric surgeons. Such studies are crucial for establishing a robust learning curve and determining the minimum number of cases necessary to attain a desirable CR when employing this surgical approach.

## Data Availability

The original contributions presented in the study are included in the article/Supplementary Material, further inquiries can be directed to the corresponding authors.

## References

[1] Springer A van den Heijkant M Baumann S. Worldwide prevalence of hypospadias. J Pediatr Urol. (2016) 12(3):152.e1–7. 10.1016/j.jpurol.2015.12.002 26810252

[2] Snodgrass WT. Tubularized incised plate (TIP) hypospadias repair. Urol Clin North Am. (2002) 29(2):285–90. 10.1016/s0094-0143(02)00045-9 12371220

[3] Snodgrass W. Tubularized, incised plate urethroplasty for distal hypospadias. J Urol. (1994) 151(2):464–5. 10.1016/s0022-5347(17)34991-1 8283561

[4] Wohl H. The cusum plot: its utility in the analysis of clinical data. N Engl J Med. (1977) 296(18):1044–5. 10.1056/NEJM197705052961806 846547

[5] Chaput de Saintonge DM Vere DW. Why don't doctors use cusums? Lancet. (1974) 1(7848):120–1. 10.1016/s0140-6736(74)92345-9 4130314

[6] Patriti A Marano L Casciola L. MILS in a general surgery unit: learning curve, indications, and limitations. Updates Surg. (2015) 67(2):207–13. 10.1007/s13304-015-0317-0 26164140

[7] Yap CH Colson ME Watters DA. Cumulative sum techniques for surgeons: a brief review. ANZ J Surg. (2007) 77(7):583–6. 10.1111/j.1445-2197.2007.04155.x 17610698

[8] Bush NC Barber TD Dajusta D Prieto JC Ziada A Snodgrass W. Results of distal hypospadias repair after pediatric urology fellowship training: a comparison of junior surgeons with their mentor. J Pediatr Urol. (2016) 12(3):162.e1–4. 10.1016/j.jpurol.2015.12.007 27317623

[9] Pippi Salle JL Sayed S Salle A Bagli D Farhat W Koyle M Proximal hypospadias: a persistent challenge. Single institution outcome analysis of three surgical techniques over a 10-year period. J Pediatr Urol. (2016) 12(1):28.e1–7. 10.1016/j.jpurol.2015.06.011 26279102

[10] Zu'bi F Chua M El Ghazzaoui A Kim JK Shiff M Rickard M Competency in tubularized incised plate repair for distal hypospadias: cumulative sum learning curve analysis of a single surgeon experience. J Urol. (2020) 204(6):1326–32. 10.1097/JU.0000000000001231 32614254

[11] Chua ME Ming JM Kim JK Degheili J Santos JD Farhat WA. Competence in and learning curve for pediatric renal transplant using cumulative sum analyses. J Urol. (2019) 201(6):1199–205. 10.1097/JU.0000000000000021 30633113

[12] Li J Li S Yang Z Ke Z Zhang T Yin J. A simple technique to repair distal and mid-shaft hypospadias using a de-epithelialized Byars’ flap. J Int Med Res. (2022) 50(8):3000605221115150. 10.1177/03000605221115150 35999815 PMC9421228

[13] Chen SC Yang SS Hsieh CH Chen YT. Tubularized incised plate urethroplasty for proximal hypospadias. BJU Int. (2000) 86(9):1050–3. 10.1046/j.1464-410x.2000.00966.x 11119100

[14] Snodgrass W Koyle M Manzoni G Hurwitz R Caldamone A Ehrlich R. Tubularized incised plate hypospadias repair for proximal hypospadias. J Urol. (1998) 159(6):2129–31. 10.1016/S0022-5347(01)63293-2 9598557

[15] Tam YH Pang KK Wong YS Tsui SY Wong HY Mou JW Improved outcomes after technical modifications in tubularized incised plate urethroplasty for mid-shaft and proximal hypospadias. Pediatr Surg Int. (2016) 32(11):1087–92. 10.1007/s00383-016-3954-6 27473011

[16] Abouzeid AA. Modified Byars’ flaps for securing skin closure in proximal and mid-penile hypospadias. Ther Adv Urol. (2011) 3(6):251–6. 10.1177/1756287211427722 22164194 PMC3229250

[17] Horowitz M Salzhauer E. The “learning curve” in hypospadias surgery. BJU Int. (2006) 97(3):593–6. 10.1111/j.1464-410X.2006.06001.x 16469033

[18] Parikh AM Park AM Sumfest J. Cumulative summation (CUSUM) charts in the monitoring of hypospadias outcomes: a tool for quality improvement initiative. J Pediatr Urol. (2014) 10(2):306–11. 10.1016/j.jpurol.2013.10.007 24290222

[19] Rompre MP Nadeau G Moore K Ajjaouj Y Braga LH Bolduc S. Learning curve for TIP urethroplasty: a single-surgeon experience. Can Urol Assoc J. (2013) 7(11–12):E789–94. 10.5489/cuaj.1376 24474999 PMC3879736

[20] Kim JK Shiff M Chua ME Zu'bi F Ming JM Pokarowski M Time to event analysis for post-hypospadias repair complications: a single-surgeon experience. World J Urol. (2021) 39(10):3913–9. 10.1007/s00345-021-03689-3 33829331

[21] Lucas J Hightower T Weiss DA Van Batavia J Coelho S Srinivasan AK Time to complication detection after primary pediatric hypospadias repair: a large, single center, retrospective cohort analysis. J Urol. (2020) 204(2):338–44. 10.1097/JU.0000000000000762 31971496

[22] Winberg H Arnbjornsson E Anderberg M Stenstrom P. Postoperative outcomes in distal hypospadias: a meta-analysis of the mathieu and tubularized incised plate repair methods for development of urethrocutaneous fistula and urethral stricture. Pediatr Surg Int. (2019) 35(11):1301–8. 10.1007/s00383-019-04523-z 31372729 PMC6800881

[23] Pfistermuller KL McArdle AJ Cuckow PM. Meta-analysis of complication rates of the tubularized incised plate (TIP) repair. J Pediatr Urol. (2015) 11(2):54–9. 10.1016/j.jpurol.2014.12.006 25819601

[24] Cheng H Clymer JW Po-Han Chen B Sadeghirad B Ferko NC Cameron CG Prolonged operative duration is associated with complications: a systematic review and meta-analysis. J Surg Res. (2018) 229:134–44. 10.1016/j.jss.2018.03.022 29936980

